# African and Asian strains of Zika virus differ in their ability to infect and lyse primitive human placental trophoblast

**DOI:** 10.1371/journal.pone.0200086

**Published:** 2018-07-09

**Authors:** Megan A. Sheridan, Velmurugan Balaraman, Danny J. Schust, Toshihiko Ezashi, R. Michael Roberts, Alexander W. E. Franz

**Affiliations:** 1 Bond Life Sciences Center, University of Missouri, Columbia, Missouri, United States of America; 2 Department of Biochemistry, University of Missouri, Columbia, Missouri, United States of America; 3 Department of Veterinary Pathobiology, University of Missouri, Columbia, Missouri, United States of America; 4 Department of Obstetrics, Gynecology and Women's Health, University of Missouri, Columbia, Missouri, United States of America; 5 Division of Animal Sciences, University of Missouri, Columbia, Missouri, United States of America; Instituut voor Tropische Geneeskunde, BELGIUM

## Abstract

Zika virus (ZIKV) drew worldwide attention when a recent epidemic was linked to fetal microcephaly. Here we used human embryonic stem cell derived trophoblasts as a model for primitive placental trophoblast to test the hypothesis that there are differences in how the two genetically distinct ZIKV lineages, African (AF) and Asian (AS), target the human placenta. Upon infection with three AF (ib-H30656, SEN/1984/41525-DAK, and MR-766) and three AS (FSS13025, MexI-44, and PANcdc259249) ZIKV strains, we observed that severe placental cell lysis was only induced after infection with AF strains, while viral replication rates remained similar between both lineages. Differences in cytopathic effects (CPE) were not observed in Vero cells, indicating that the AF strains were not inherently superior at cell lysis. Taken together, we propose that infection with AF strains of ZIKV early in pregnancy would likely result in pregnancy loss, rather than allow further fetal development with accompanying brain damage. Our results also suggest that the long term laboratory-adapted MR-766 strain does not behave aberrantly in cell culture relative to other AF lineage strains.

## Introduction

The mosquito-borne Zika virus (*Flaviviridae*; *Flavivirus*; ZIKV) is the most recently emerging arthropod-borne virus that has infected human populations at an epidemic scale in the western hemisphere [1]. Before its introduction into the Americas, which may have occurred as early as 2013 and caused by human activity [2], the virus was known to be prevalent in Africa and Asia. In tropical regions of these two continents, the virus was believed to circulate predominantly in sylvatic transmission cycles between Aedine mosquitoes and non-human primates, which acted as natural reservoirs, with occasional outbreaks occurring among the local human population [3]. ZIKV was first isolated from a sentinel rhesus monkey in the Zika forest of Uganda in 1947 [4]. A major ZIKV epidemic was recorded for the first time on Yap Island, Federated States of Micronesia, in 2007 with another major epidemic following in French Polynesia in 2013 [5, 6].

Typical ZIKV disease symptoms included self-limiting febrile illnesses with fever, rash, headache, and occasionally arthritis [1]. Thus, disease manifestations caused by ZIKV infection appeared to be similar to those caused by other mosquito-borne arboviruses such as dengue virus (DENV) and chikungunya virus (CHIKV). However, during the epidemic ZIKV outbreaks in French Polynesia and following its emergence in Brazil, atypical disease symptoms such as the Guillain Barre Syndrome among adults and microcephaly among fetuses and infants of infected pregnant women became apparent, indicating changes in viral tropism and/or virulence during these outbreaks [7–9]. Furthermore, the risk of fetal microcephaly appeared to be mainly associated with first trimester infections [6, 10–13].

Genetically, ZIKV isolates have been clustered into two major lineages (= genotypes), African (in this study designated AF) and Asian (designated AS), with the AF lineage being ancestral to the AS lineage. Viruses of both lineages vary by less than 4% in their amino acid sequences [14, 15]. In contrast to contemporary AS strains of the virus, AF ZIKV strains have not been associated with fetal birth defect-like symptoms. Many groups have suggested possible reasons why the more recently described, contemporary AS strains of ZIKV suddenly became capable of producing epidemic outbreaks and adverse outcomes during pregnancy. One possibility is that the AS strains are more efficiently transmitted by the highly anthropophilic mosquito vector, *Aedes aegypti*, thereby accelerating geographical spread of the disease. However, evidence supporting this explanation has been mixed. In two studies, AF strains outcompeted contemporary AS strains in mosquito cell cultures (C6/36 and Aag2) [16, 17], while in another, contemporary AS strains were able to infect *Ae*. *aegypti* mosquitoes more efficiently than an older AS strain (FSS13025) [18].

An alternative explanation for the greater virulence of contemporary AS strains is that they are able to infect and replicate in their human target cells more rapidly than the AF strains. However, AF ZIKV strains have been observed to infect human and mouse neuronal stem cells [19–22], dendritic cells [23], brain organoids [24, 25] and the central nervous system in mice [26] at least as efficiently as the AS strains implicated in fetal microcephaly. Finally, it is possible that contemporary AS strains cross the placenta and, subsequently, the blood brain barrier of the fetal brain more efficiently than AF ZIKV strains. To this point, a single serine to asparagine mutation (S139N) within the prM-encoding region of the genomes of three contemporary AS ZIKV strains (GZ01, SZ01, and MRS_OPY_Martinique_PaRi_2015) led to enhanced infectivity in both human and mouse neural progenitor cells and, additionally, to higher rates of microcephaly in the fetuses of infected mice when compared to an older AS strain (FSS13025) [27]. It needs to be emphasized that both AF and AS ZIKV can infect fetal brains in pregnant immunodeficient mice, leading to central nervous system disorders [26, 28, 29]. Despite all these observations, it still remains unclear why AF ZIKV strains have not been associated so far with fetal microcephaly or other adverse pregnancy outcomes. Furthermore, researchers agree that ZIKV outbreaks in Africa may be underreported as the clinical disease symptoms of ZIKV often resemble those of DENV or CHIKV and precise diagnostic tools are not easily available in rural settings [30].

Our laboratory hypothesized the absence of fetal microcephaly as a disease manifestation associated with AF strain infections might reflect the placenta’s role in protecting the fetus against pathogens as opposed to a differential susceptibility of fetal neuronal cells and their progenitors to ZIKV infection. The syncytiotrophoblast that forms the outer layer of the villous structures of the mature human placenta is the principal barrier between the fetus and maternal blood. At the end of gestation (term) this multinucleate cell layer is known to be resistant to infection by a variety of viruses including ZIKV [31–33]. Little is known about the response of trophoblast to ZIKV within the first trimester of pregnancy. However, the fetus appears to be particularly vulnerable to other viral infections like Rubella (*Togaviridae*, *Rubivirus*) and Cytomegalovirus (*Herpesviridae*) in early pregnancy [34–37]. Previously, we showed that undifferentiated human embryonic stem cells lines (ESC) were resistant to infection with AS (FSS13025 Cambodia) and AF (MR-766 Uganda) ZIKV [38]. In striking contrast, ESC became highly susceptible to ZIKV infection upon their differentiation into trophoblast driven by a combination of BMP4 and signaling inhibitors of FGF2 (PD173074) and TGFB/ACTIVIN (A83-01). These ESC-derived trophoblast (ESCd) have been postulated to represent the primitive placental trophoblast encountered in the implantation stage of development before the formation of mature chorionic villi [39–41]. We found that ESCd are rapidly lysed by the purportedly more benign AF Uganda ZIKV, while the AS Cambodia strain associated with more severe pregnancy-related clinical disease infected these cells but caused only minimal cell lysis. A caveat to this preliminary finding is the recognition that the AF Uganda strain used in our work is highly mouse brain-adapted, which could have resulted in a loss or change of its original virulence determinants [14]. Here we expand on our previous study by analyzing the infection patterns of a group of more genetically diverse AF and AS ZIKV strains in ESCd cultures to assess whether clear differences between AS and AF viruses could be identified in this host system.

## Methods

### Propagation of ZIKV in Vero cells

Vero cells (ATCC; CCL-81) were seeded into T25 flasks (5x10^5^ cells/flask). After 2–3 days (~70% confluent density ~2x10^6^ cells/flask) the monolayer was infected with ZIKV (FSS13025 Cambodia, GenBank # KU955593.1; ib-H30656 Nigeria, GenBank # KU963574.2; MexI-44 Mexico, GenBank # KX856011; MR-766 Uganda, GenBank # HQ234498.1; PANcdc259249 Panama, GenBank # KX156775; or SEN/1984/41525-DAK Senegal, GenBank # KU955591.1) at a multiplicity of infection (MOI) of 0.01. Flasks were incubated at 37°C for 1 h, with gentle rocking. After adding 5 ml additional medium (DMEM, Thermo Scientific, supplemented with 10% v/v FBS) cultures were maintained for a further 72–120 h. ZIKV stocks were generated by collecting the medium at 72–120 h post infection (PI), when typically, 40–50% of cells showed cytopathic effects (CPE). Virus stocks were maintained at -80°C until used. All stocks were aliquoted and then titered by plaque assay. All infection experiments were performed with ZIKV stocks that had only undergone a single passage in Vero cells. The Panama strain of ZIKV (PANcdc259249) was obtained from BEI Resources, (NIAID-NIH, NR-50220). All other viruses were obtained through the UTMB-Galveston Arbovirus Reference Collection maintained at the World Reference Center for Emerging Viruses and Arboviruses (WRCEVA).

### Determination of ZIKV titers by plaque assay

Vero cells were plated at a density of 5x10^4^ cells/well in a 24 well plate and cultured for one day at 37°C and 5% CO_2_/air. After removing the medium, viral supernatant (150 μl; 10^−1^–10^−6^ dilutions) was added to each well. The culture plates were rocked every 15 min for 1 h before 1ml/well agarose/nutrient solution overlay [1% agarose, 1x Medium 199 (Sigma-Aldrich, St. Louis, MO), 10% FBS, 4% NaHCO_3_, 0.5% MEM vitamins, 0.5% MEM amino acids (Mediatech Inc., Manassas, VA)]. Plates were incubated for 5 days at 37°C and 5% CO_2_/air. Cells were fixed with 0.5 ml of 10 v/v % formalin for 5 h before removing the agarose. Cells were then stained with 0.2% crystal violet solution (0.2% w/v gentian violet, 20% v/v ETOH) to visualize the plaques (S2 Fig).

### Human ESC culture and differentiation

Human ESC (H1, WA01) were cultured in six-well tissue culture plates (Thermo Scientific) coated with Matrigel (BD Bioscience) under an atmosphere of 5% CO_2_/air at 37°C in mTeSR1 medium (STEMCELL Technologies). Cells were passaged every 5–6 days. The method for trophoblast differentiation has been described elsewhere [42]. Briefly, the day after passaging onto Matrigel coated dishes at 1.2x10^4^ cells/cm^2^, the culture medium was changed to DME/F12 medium (Thermo Scientific) with knock-out serum replacement (KOSR, Invitrogen) that had been conditioned by mouse embryonic fibroblasts (MEF) and supplemented with FGF2 (4 ng/ml). After 24 h, the conditioned medium was replaced with daily changes of non-conditioned DME/F12/KOSR medium lacking FGF2, but containing BMP4 (10 ng/ml), A83-01 (1 μM) and PD173074 (0.1 μM) (BAP treatment) for up to 7 days. Control cultures (ESCu) were maintained in conditioned medium containing 4 ng/ml FGF2.

### JAr and Vero cell culture

JAr cells (ATCC, HTB-144) and Vero cells (ATCC, CCL-81) were cultured in DMEM (Thermo Scientific) supplemented with 10% v/v FBS. To passage cells, the monolayer was washed with 1x DPBS twice, and then incubated with either Trypsin or TrypLE (Thermo Scientific) for ~4 min at 37°C.

### Crystal violet staining and imaging

Colonies were fixed in methanol for 5 min and then stained with crystal violet solution (0.2% w/v) for 5 min and rinsed with tap water before imaging with a Leica M205 FA stereomicroscope with a Leica DFC 7000T high sensitivity color camera. Variability in the images (brightness, shadows, and background color) are due to the reflection/refraction patterns elicited by the microscope light through the plastic cell culture dish.

### Immunostaining

Cells were grown on coverslips coated with Matrigel in six-well tissue culture plates. After fixing the cells in 4% v/v paraformaldehyde in PBS for 12 min and permeabilizing in 1% Triton X-100/PBS, coverslips were placed in 5% w/v bovine serum albumin in PBS for 1 h. Cells were then incubated overnight at 4°C with polyclonal primary anti-ZIKV antibodies (SAB-153 Biotherapeutics), diluted 1:2000 in 5% w/v bovine serum albumin in PBS. Secondary antibody staining was performed with Alexa Fluor 568 goat anti-human monoclonal Ab (Life Technologies), diluted 1:300 in PBS. Images were captured under a Zeiss Axiovert 200M wide-field microscope equipped with a fluorescent imaging system and an ORCA-ER camera.

### ZIKV growth curves

ESC were plated onto 12-well tissue culture dishes (2.5x10^4^ cells/well) and subsequently treated with BAP for 4 days to generate trophoblast cells. JAr and Vero cells were plated onto 12-well tissue culture dishes ~5 h prior to infection (7.5x10^4^ cells/well). All cells were infected with ZIKV at an MOI of 0.1. The initial input of virus added to each well was used as a 0 h time point. After 1 h of viral absorption, an additional 750 μl of fresh culture medium was added. Samples of infected cell supernatants were collected at each designated time point (8, 16, 24, 32, 40 and 48 h PI) and stored at -80°C before titration by plaque assay on Vero cells. Growth curves were performed in triplicate. Data presented are from one representative experiment.

### Cell cytotoxicity assay

Cell viability was measured by using a Colorimetric Cell Cytotoxicity Assay Kit (Abcam) according to manufacturers’ instructions. Briefly, ESCd were cultured in 48-well culture dishes and infected with 1 MOI of each ZIKV strain. Cell viability was measured 48 h and 60 h PI and normalized to mock infected values.

### Statistics

Data are presented as means ± SEM. Comparisons of viral titers were performed by using a repeated measures two-way ANOVA (GraphPad Prism) with Bonferroni post-tests at each time point. Significance was defined as *p* < 0.05. For the cell viability assays, comparisons between the mock infected controls and each viral strain were performed by using a one-way ANOVA with Bonferroni post-tests. Each time point (48 and 60 h PI) was assessed separately because they were completed as two independent experiments. Values of *p ≤ 0*.*05* are considered to support the conclusion that differences are significant.

## Results

### AF strains of ZIKV exert more rapidly-destructive CPE on stem cell-derived trophoblast than AS viruses

**Fig 1:**
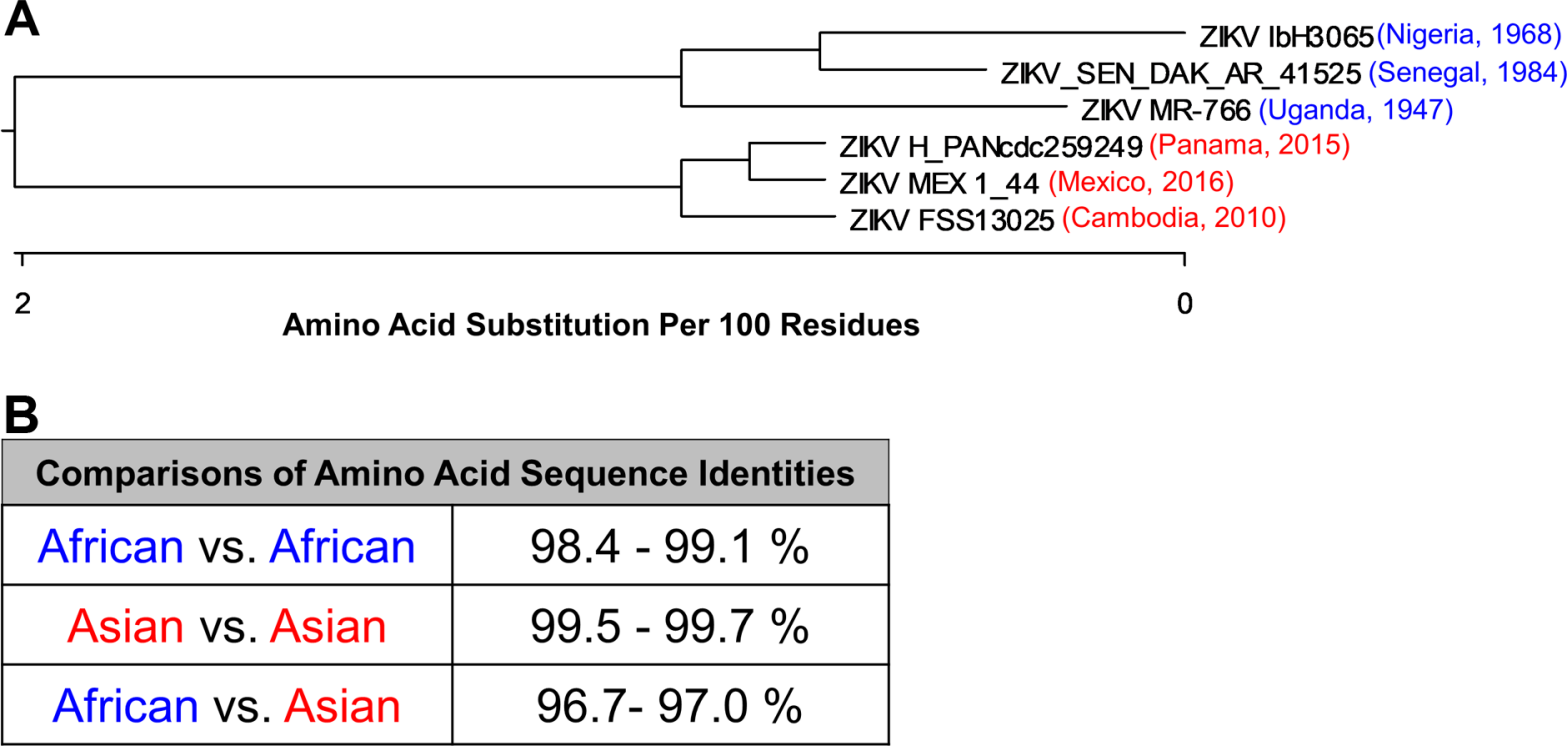
Phylogenetic tree for genetic comparison of the ZIKV strains used in this study. (A) Published amino acid sequences (NCBI GenBank) of the six ZIKV strains were used to construct a phylogenetic tree using the neighbor-joining method. The AS (red) and AF strains (blue) show a clear separation into two distinct clusters based on their amino acid sequences. (B) Comparison of amino acid sequence identities between the six viruses when clustered into the AF and AS lineages.

We showed previously that ESC derived trophoblast cells (ESCd) were highly susceptible to infection with MR-766 (Uganda), an AF ZIKV, and that infection with this virus induced severe CPE in ESCd. Conversely, when ESCd were infected with the AS strain FSS13025 (Cambodia), the cells were susceptible to infection, but infection resulted in weak CPE. To further evaluate whether viral induction of rapid and widespread cell lysis was an inherent property of the AF ZIKV strains, we included four additional viral strains (with low passage history, S1 Table) in the analysis: two AF strains, ib-H30656 (Nigeria) and SEN/1984/41525-DAK (Senegal), and two AS strains, MexI-44 (Mexico) and PANcdc259249 (Panama). A phylogenetic tree was constructed by using the neighbor joining method, based on the full-length amino acid sequences of the six virus strains (Fig 1A). The viruses were separated into two major clusters according to their lineages (AF or AS). Further, the tree confirmed that within each major cluster, the strains Uganda and Cambodia were genetically more distinct from the other two viruses of each respective cluster. Overall, the amino acid sequences of the AF and AS viruses differed by approximately 3% (Fig 1B).

**Fig 2:**
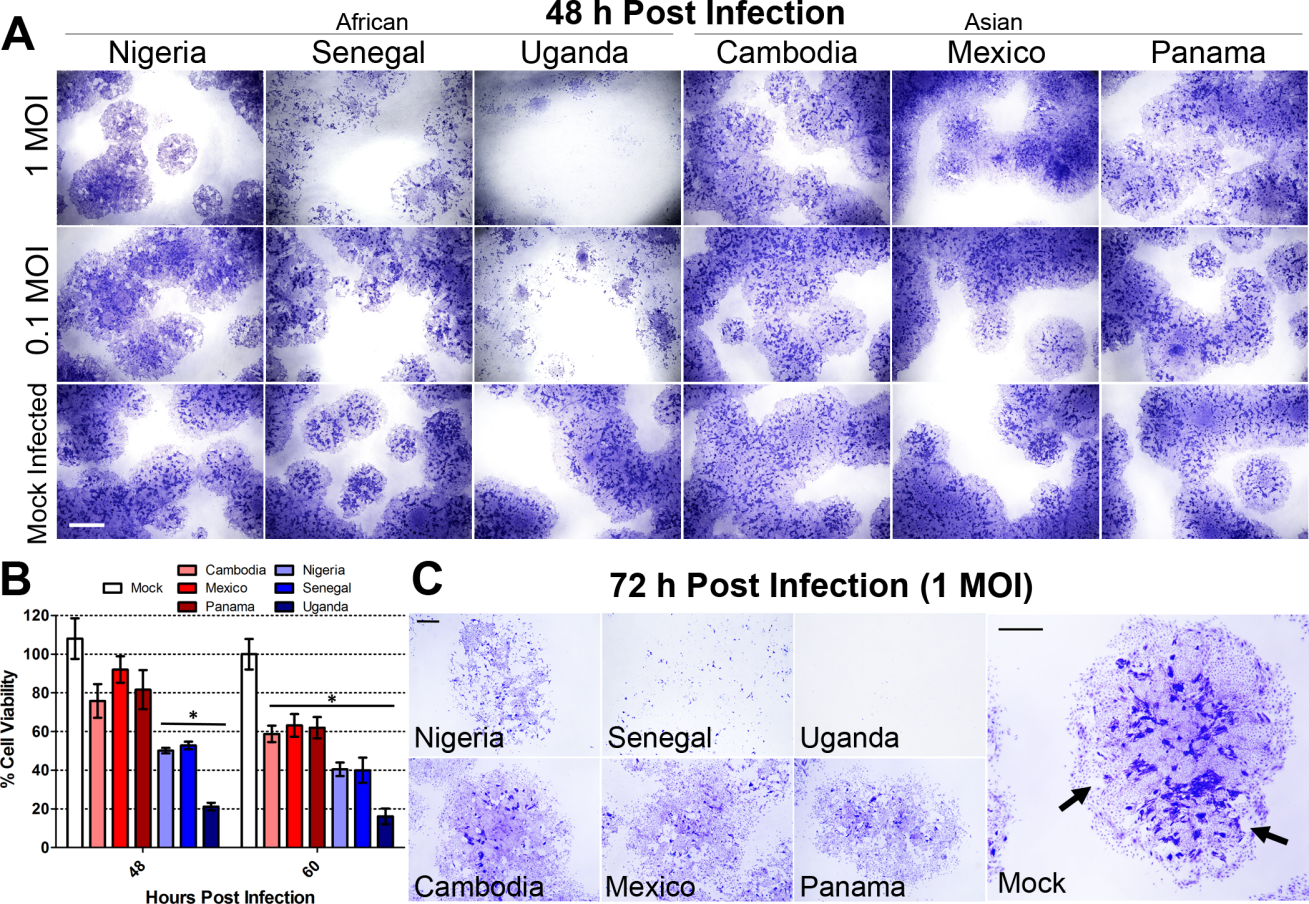
Cytopathic effects (CPE) caused by three AF and three AS ZIKV strains in ESCd at 48, 60 and 72 h PI. Colonies of ESC were grown in 6-well matrigel-coated cell culture dishes and differentiated for 4 days by BAP treatment. After 4 days, cells were infected with each ZIKV strain at 1, 0.1 or 0 (mock) MOI. (A) At 48 h PI, cells were fixed and stained with crystal violet to observe CPE. Cell lysis was evident in cells infected with all three of the AF strains, even at 0.1 MOI, whereas minimal cell lysis was evident in AS ZIKV infected (1 MOI) colonies. (B) Cell viability was measured at 48 h and 60 h PI to demonstrate the extent of lysis induced by each ZIKV strain at 1 MOI. At 48 h post- infection, only the three AF strains of ZIKV (shades of blue) showed a significant reduction in cell viability when compared to the mock infected control (one-way ANOVA, *p* < 0.001). Conversely, none of the AS strains of ZIKV (shades of red) induced a significant reduction in cell viability. All six ZIKV strains showed a significant reduction in cell viability when compared to the mock infected control at 60 h PI (*p* < 0.01 (Mexico), *p* < 0.001 (all other strains)). Data are presented as mean ± SEM (n = 5). (C) The extent of cell lysis at 72 h PI is accentuated by images taken at a higher magnification. At 72 h PI, severe cell lysis was evident in cells infected with all three AF strains and became apparent in cells infected with the AS strains. In the images, areas of deep purple staining highlight the areas of multinucleated syncytia. There were no syncytial areas present in the cultures infected with the AF strains of ZIKV, while small areas remained visible after infection with the AS strains. At 72 h PI, mock infected controls showed non-virus induced cell lysis, as the cells naturally started to die off at this time point post-differentiation. These areas are indicated by black arrows. Scale bars are 3 mm (A) and 1 mm (C).

To assess cytopathic differences elicited by ZIKV infection, ESC (H1, WA01) were seeded into 6-well tissue culture dishes and directed along the differentiation pathway to trophoblast by BAP treatment. On the fourth day of differentiation, cells were infected with each ZIKV strain at a MOI of 1, 0.1 and 0 (mock infected). Cells were fixed and stained with crystal violet, a dye that is particularly effective at demarking areas of emerging syncytiotrophoblast 48 h and 72 h PI in order to visualize the extent of colony destruction. At 48 h PI there was little evidence for cell damage by any of the AS ZIKV infections at either MOI, but areas of marked cell lysis were evident with all three AF strains of the virus (Fig 2A). This outcome is consistent with our previous findings comparing only the Uganda and Cambodia strains. The AF Nigerian ZIKV induced more colony damage than any of the AS strains, but was less destructive than the AF Uganda and Senegal strains. Cell viability was quantified after 48 h and 60 h of ZIKV infection to demonstrate the progression of cell lysis over time (Fig 2B). Compared to the mock infected controls, a significant decrease in cell viability was only observed after infection with any of the three AF strains at 48 h PI. (*p* < 0.001). There was no significant difference in cell viability between the three AS strains or mock infected controls at 48 h PI. By the 60 h time point, lysis was clearly induced by all six ZIKV strains, albeit more severe after infection with any one of the three AF strains (*p* < 0.01). The AF Uganda strain showed the highest degree of lysis since the majority of the cells were lysed after just 48 h of infection. A similar scenario was observed at 72 h PI in that the CPE continued to progress after infection with the AF strains. However, following infection with any of the AS ZIKV strains, evidence of cell lysis was just beginning to appear at 72 h PI (Fig 2C). Under high magnification, areas of syncytium, recognizable by intense purple staining, were nearly absent in ESCd cultures infected with any of the three AF strains of ZIKV, whereas small areas of syncytium were still evident after infection with any of the three AS ZIKV strains (Fig 2C). It should be noted that at 72 h PI, seven days after initiating the BAP differentiation protocol, some areas of syncytium in the control (mock-infected colonies) were beginning to loosen from the substratum, i.e. colony damage unrelated to viral infection could be detected (indicated by black arrows in Fig 2C). This phenomenon invariably accompanies BAP-directed differentiation of ESC after about a week of differentiation and leads to a fall in human chorionic gonadotropin (hCG) production by the cultures [42]. To ensure that the ESCd had become infected with the AS and AF strains of ZIKV, the presence of virus within the cells was confirmed by immunohistochemistry using trans-chromosomic bovine-derived anti-ZIKV human polyclonal antibodies (SAb Therapeutics) (S1A and S1B Fig). The intensity of infection at 24 h PI was similar among all ZIKV strains except Uganda (*p* < 0.001), suggesting that the lytic differences were not simply a consequence of initial virus replication efficiencies.

### Relative susceptibility of JAr cells to the AF and AS strains of ZIKV

**Fig 3:**
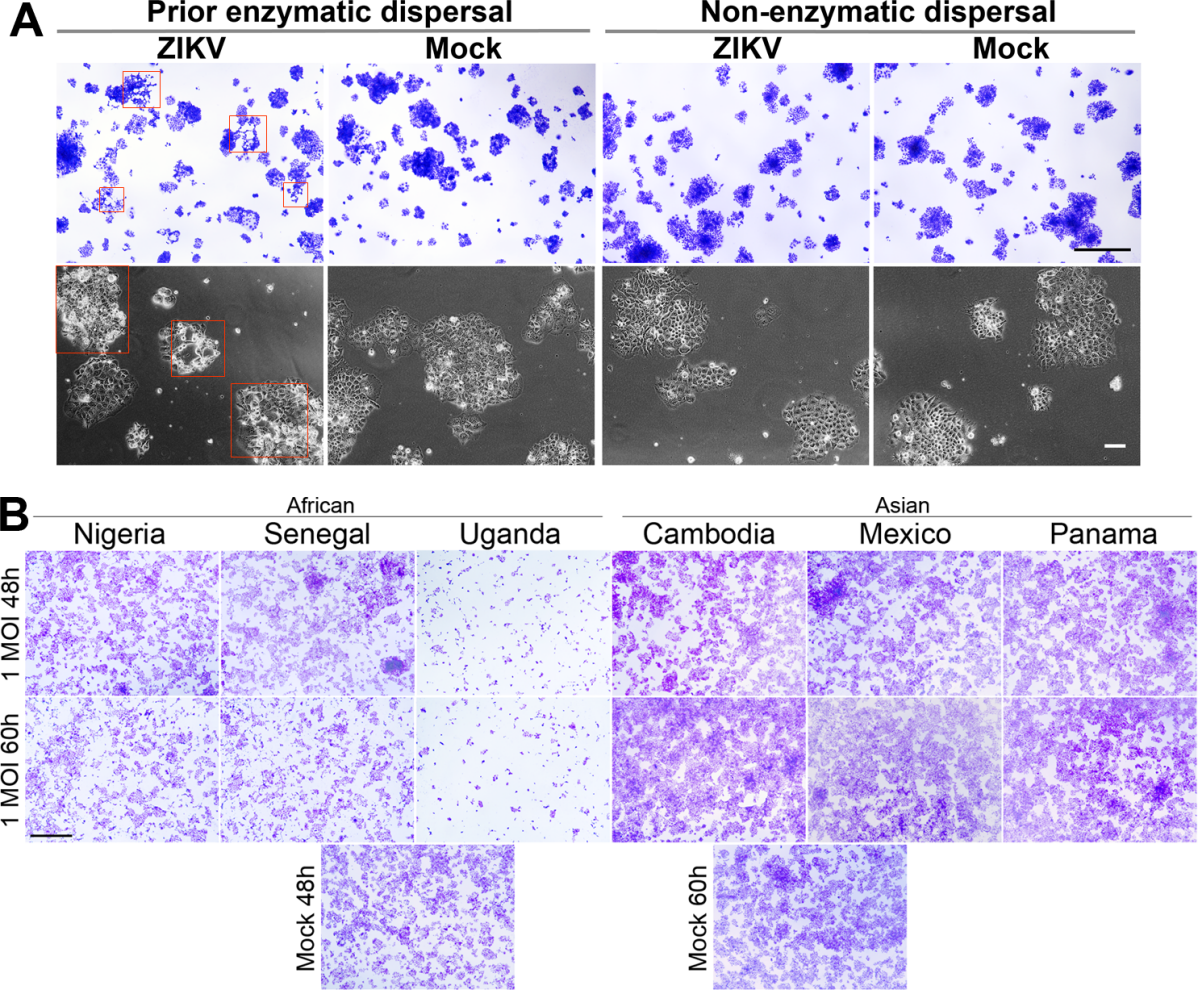
Cytopathic effects (CPE) caused by three AF and three AS ZIKV strains in JAr cells. (A) Two separately cultured groups of JAr cells were exposed continuously to either enzymatic dispersal (>10 passages, trypsin) or non-enzymatic dispersal (gentle disassociation reagent, STEMCELL Technologies). Prior to infection, each group was dispersed by a non-enzymatic agent, to allow uniform cell dispersal. Two days after plating, the groups were infected with the AF Uganda strain of ZIKV at an MOI of 0.27. At 48 hours PI, live cell images were captured to demonstrate CPE (bottom panel). Cells were fixed and stained with crystal violet (top panel). Areas showing clear CPE are highlighted by red rectangles. (B) JAr cells were infected with each ZIKV strain at 1 MOI and fixed either at 48 h or 60 h PI. Respective mock infected controls are shown below. Lysis is only evident after infection with the three AF strains of ZIKV. Scale bars are 100 μm for live cell images in (A) and 1 mm in (A) and (B) for crystal violet stained images.

JAr cells (ATCC/HTB-144) were originally derived from a choriocarcinoma and are considered to be of trophoblast origin [43]. They have previously been used to assess ZIKV susceptibility in several laboratories, including ours [32, 38, 44, 45]. Here we examined their comparative susceptibility to the six ZIKV strains. A specific goal was to test whether the observed lytic infection of trophoblast cells at low MOI was a characteristic feature of the AF strains. In our initial experiments [38], JAr cells were passaged by using Gentle Disassociation Reagent (STEMCELL Technologies), which is free of proteolytic enzymes. As shown in Fig 3A, JAr cells passaged in this manner appeared to be protected from the severe CPE of the Uganda strain (Fig 3A, “non-enzymatic dispersal”). By contrast, cells that had been serially passaged by means of trypsin treatment ("prior enzymatic dispersal") showed the presence of strong CPE as indicated by the red rectangles in Fig 3A.

Similarly, cells that had been serially passaged by means of trypsin treatment were readily lysed following infection with the Senegal or Nigeria strains of ZIKV (Fig 3B). Conversely, even after switching from non-enzymatic dissociation to trypsin dissociation in order to passage JAr cells, none of the AS ZIKV were able to elicit cell lysis. Even at 72 h PI, there were no indications of cell death with any of the AS strains (S2 Fig). Our data indicate that both trophoblast cell types, JAr and ESCd, were differentially susceptible to AF and AS ZIKV strains, since JAr cells were lysed by AF but not by AS viruses, while ESCd were more rapidly lysed by AF strains than by AS strains.

### Relative susceptibility of Vero cells to AF and AS ZIKV strains

**Fig 4:**
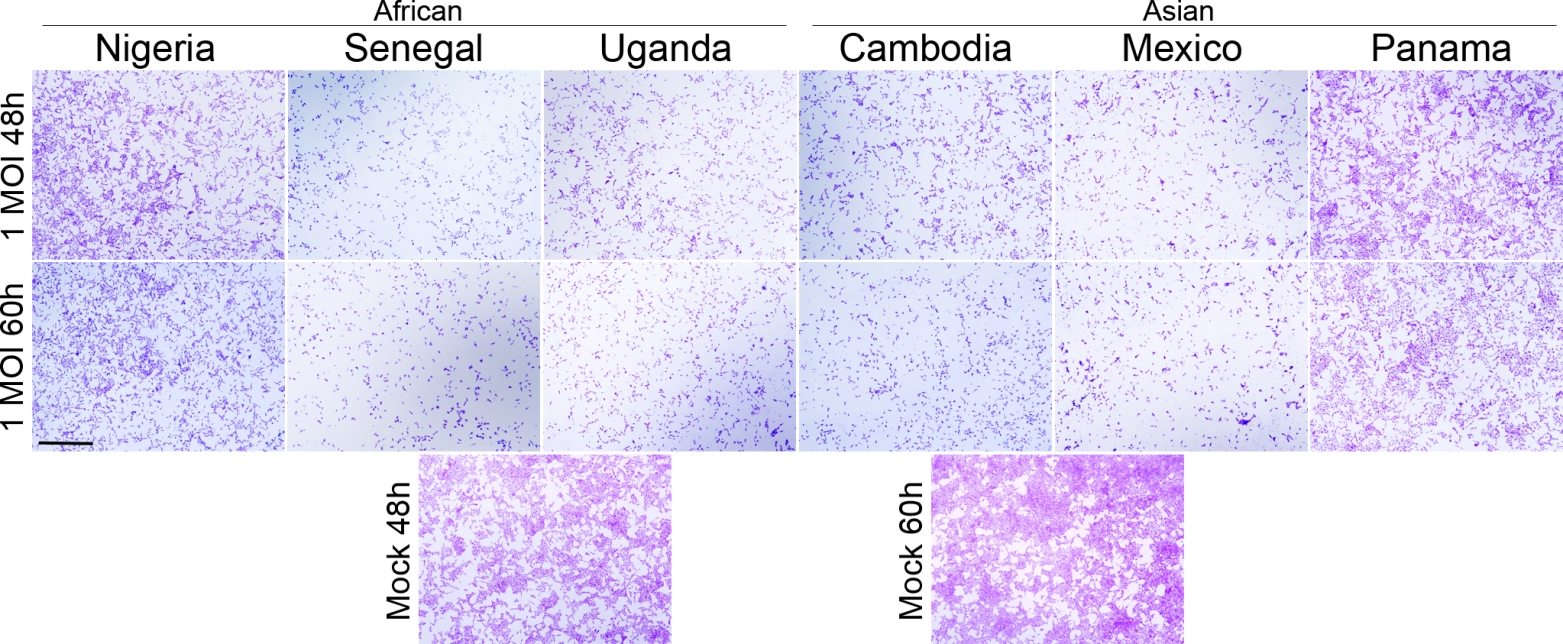
Cytopathic effects (CPE) caused by three AF and three AS ZIKV strains in Vero cells. Vero cells were infected with each ZIKV strain at 1 MOI and fixed either at 48 h or 60 h PI. Respective mock infected controls are shown below. Similar CPE are evident after infection with all AF and AS ZIKV strains, although the AF Nigeria and AS Panama strains elicited weaker CPE when compared to the other strains. Scale bar is 1 mm.

We next asked whether an inherent ability to induce severe cell lysis, independent of the infected host cell type, is a general property of the AF ZIKV strains. To address this question, we used Vero cells (*Cercopithecus aethiops* kidney cells, ATCC/CCL-81), a host cell type of primate origin that has not been associated with ZIKV pathogenesis. Vero cells were infected with ZIKV at an MOI of 1 or 0. The cells were fixed and stained with crystal violet at 48, 60, and 72 h PI. Interestingly, both AF and AS ZIKV caused similar CPE in Vero cells during their infection, although the AF Nigeria and AS Panama strains elicited somewhat weaker CPE, i.e. cell death, than the other four ZIKV strains (Fig 4; S2 Fig). Thus, the AF and AS strains exhibited similar levels of virulence in Vero cells, but clearly showed different infection phenotypes in cell types representing human trophoblasts, where AF viruses were invariably more destructive than the AS ZIKV strains.

### Growth curve analyses of AF and AS ZIKV strains in ESCd, JAr, and Vero cells

**Fig 5:**
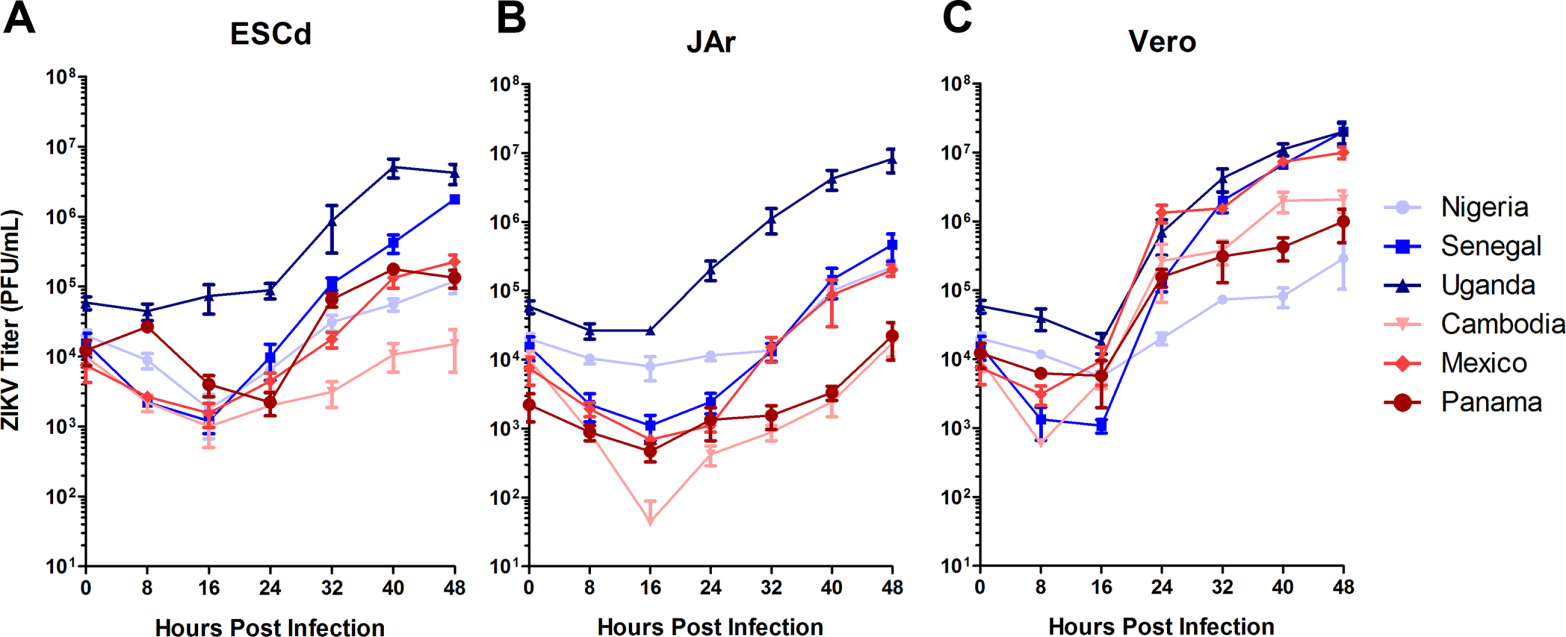
Comparative growth curve analyses of three AF and three AF ZIKV strains in ESCd, JAr, and Vero cells. Cells were infected with the six ZIKV strains at a MOI of 0.1. Cell supernatants were harvested at the indicated time points for titration by plaque assay in Vero cells. Growth curve analyses were performed in triplicate and plotted as SEM. Growth curves representing the AF strains are highlighted in shades of blue and those representing the AS strains are shown in shades of red. (A) In ESCd, significantly higher virus concentrations were observed by 48 h PI for the AF Uganda and the AF Senegal strains when compared to all other strains (*p* < 0.05). No significant differences were observed between the Nigeria, Cambodia, Mexico, and Panama strains. (B) In JAr cells, significantly higher concentrations were observed by 48 h PI for the AF Uganda strain when compared to all other strains (*p* < 0.001). No significant differences were observed between the Senegal, Nigeria, Cambodia, Mexico, and Panama strains. (C) In Vero cells, significantly higher virus concentrations became obvious by 48 h PI for the AF Uganda, the AF Senegal, and the AS Mexico strains when compared to the other three viruses (*p* < 0.01). No significant differences were observed between the Nigeria, Cambodia and Panama strains.

Once it became obvious that there were phenotypic differences between AF and AS ZIKV strains following infection of ESCd, JAr and Vero cells, we sought to determine whether this was simply a reflection of differences in the relative replication rates of the viruses. To address this question, we conducted growth curve analyses by infecting each cell type with a ZIKV strain at a MOI of 0.1. Immediately after virus infection, a sample of the cell culture medium was collected and used as the 0 h time point. Additional cell culture medium samples were removed at 8, 16, 24, 32, 40 and 48 h PI. The quantity of infectious virus in each medium sample was then determined by plaque assays performed on Vero cells. Fig 5A–5C show the growth curves for each ZIKV strain grouped by target cell type (ESCd, JAr, and Vero cells, respectively), while S3 Fig shows the same data grouped by virus strain. In ESCd, the AF Uganda and Senegal strains produced significantly higher virus titers by 48 h PI than the other four ZIKV strains (*p* < 0.001, *p* < 0.05 respectively; Fig 5A). The three non-lytic AS strains and the lytic AF Nigeria strain showed no significant differences in their ability to replicate in ESCd. This result indicates that the ability of ESCd to support viral replication was not fundamentally responsible for the different cell lytic abilities of the AF and AS strains towards ESCd (Fig 2A and 2C). In particular, the AF Nigeria strain elicited a significant reduction in cell viability by 48 h PI in the ESCd, while the three AS strains did not (Fig 2A and 2B), yet there were no significant differences in viral titers at this same time point (Fig 5A). A similar pattern was noted in JAr cells, except that the AF Uganda strain produced significantly higher viral titers than all other ZIKV strains, including the AF Senegal strain (*p* < 0.001) (Fig 5B). No significant differences were observed between the other five strains, once again indicating that the ability of JAr cells to support viral replication was not inherently different between AS and AF ZIKV strains. Thus, viral replication dynamics of the different virus strains in trophoblast cells may in fact play a role, but, clearly, this cannot be seen as the only factor accounting for the differences in host pathology caused by the two different ZIKV lineages.

Viral replication in Vero cells displayed a pattern similar to that in ESCd; the AF Uganda and Senegal strains produced significantly higher titers than the other four strains (*p* < 0.001) (Fig 5C). The AS Mexico strain also produced significantly higher titers than the other two AS and the AF Nigeria strains (*p* < 0.01). Of note, the plaque sizes generated by all ZIKV strains, aside from the AF Nigeria strain, were similar in size (S4 Fig). From this we can speculate that overall viral fitness was lower in the AF Nigeria ZIKV strain in comparison to the other viruses. Importantly, even though the Nigeria strain showed a comparatively weak replication dynamic in all cell types, it was able to elicit CPE in both placental cell types (ESCd and JAr) more readily than the three AS ZIKV strains (Fig 2A and 2C; Fig 3B). This outcome clearly shows that replication efficiency *per se* cannot be the major factor underlying the phenotypic differences between AF and AS ZIKV strains.

## Discussion

In this study, we compared infection phenotypes and replication efficiencies of AF lineage ZIKV strains with those of the AS lineage in human placental trophoblasts and primate epithelial kidney cells to elucidate whether or not fetal birth defects might be tightly associated with the specific ZIKV genotype causing the infection. Induction of microcephaly in fetuses of infected pregnant women has been attributed to be specific to highly virulent, contemporary AS ZIKV strains, whereas the absence of birth defects following infection with AF viruses has been ascribed to a lower level of virulence of the latter [46]. Unexpectedly, however, AF strains have often been observed to cause more severe infection phenotypes, including fetal mortality, in rodent models and chicken embryos, whereas infection with the contemporary AS strains has led to central nervous system abnormalities in fetuses [17, 26, 28, 47]. Although rodent models have contributed considerably to our current knowledge of ZIKV transmission during pregnancy, these studies cannot be directly linked to human pathogenesis because immunocompetent wild-type mice and hamsters are not susceptible to ZIKV infection. Consequently, because ZIKV and DENV do not efficiently antagonize type I interferon (IFN) signaling in mice as they would do in humans, animals were treated with a single dose of blocking monoclonal antibody against mouse IFNAR1 to facilitate ZIKV infection and dissemination [48]. Recently, however, as an alternative to immunosuppression, human *STAT2* was introduced into the mouse *Stat2* locus (hSTAT2 KI) in order to obtain an immunocompetent mouse model for ZIKV pathogenesis [47]. Following subcutaneous inoculation of pregnant (hSTAT2 KI) mice, a mouse-adapted ZIKV variant derived from the AF Dakar 41519 strain [49] spread to the placenta and the fetal brain. However, it remains to be seen whether this immunocompetent model is suitable to conduct AF and AS strain comparisons in order to elucidate novel mechanisms underlying placental infection and pathology caused by the viruses.

Aside from the particular ZIKV strain, the gestational stage of the mother during ZIKV infection seems to strongly affect the ultimate disease outcome. Several mouse studies indicated that ZIKV pathogenesis can only be observed in the fetus if the mother had been infected before embryonic day 10 [29, 50, 51]. This timing may be significant because the mouse hemochorial placenta only begins to develop after embryonic day 8.5 and only approaches structural maturity by day 10.5 [52]. Epidemiological studies in humans also suggest that timing is important, with the risk of fetal microcephaly predominantly associated with first trimester infections [6, 10–13, 53]. Taken together, these studies suggest that the maturity of the placenta may play a major role in ZIKV’s ability to induce adverse pregnancy outcomes. Since we cannot directly study ZIKV transmission across the human placenta in vivo, particularly in the earliest stages of pregnancy, it is imperative that researchers develop appropriate model systems.

Our ESCd model allows the analysis of ZIKV pathogenesis during human pregnancy in vitro, including the comparative analysis of infection patterns caused by diverse strains of the virus belonging to the AF and AS lineages. These ESCd have been postulated to resemble the primitive placental trophoblast encountered during the implantation stage of development, at a time when the human conceptus might be particularly vulnerable to ZIKV infection [38, 39, 41] as suggested by outcomes observed with other viral infections during pregnancy [34–37]. The second host cell type we chose for ZIKV infections was the JAr cell line, which has been widely used as a model for first trimester trophoblast. JAr cells were originally derived from a heterogeneous population of choriocarcimoma cells [43], produce placental hormones [54, 55], and express the Class I Antigen, HLA-G [56], although their precise ontology is unclear. In particular, hierarchical clustering [57] and principle component analysis [58] of JAr cells indicate that they are distinct from primary villous cytotrophoblasts and extravillous trophoblast. The third host cell type chosen for ZIKV infections was Vero cells, which are commonly used to propagate and titer viruses due to the absence of the type I interferon gene locus [59]. Our findings indicate that the AF and AS strains of ZIKV replicate similarly and elicit equivalent levels of cell lysis in Vero cells. This suggests that the ability of the host cell to mount an interferon response is likely a mediator of the viral induced cell lysis. Therefore, it would be interesting to test whether the virus induced interferon responses in ESCd and JAr cells are different following an infection with an AS strain relative to an AF strain. The latter, for example, elicited a delayed induction of an antiviral responses by an AF (HD 78788) strain relative to an AS (H/PF/2013) strain in primary human astrocytes [60]. It is plausible to assume that this delay in the antiviral response could lead to potential differences in the extent of lysis exhibited in that cell type. On the other hand, a more robust induction of type I and type II interferons and inflammatory cytokines was displayed in the brain of STAT2 deficient mice after infection with AF strains (MR-766, DAKAR 41519) when compared to AS strains (P6-740, FSS13025, PRVABC59) [26]. Future research efforts should focus on a comparative analysis of host immune responses in ESCd, JAr, and Vero cells following AF and AS strain infection, such as examining host cell innate immune responses and conducting comparative transcriptome analysis. For example, AF and AS ZIKV strains produce different immunoprofiles in human CD14+ monocytes following blood infection [61].

As we anticipated, all three AF strains elicited more severe CPE on both ESCd and JAr cells than the three AS strains tested. Of the three AF strains, the laboratory adapted Uganda (MR-766) strain exerted by far the most damaging lytic effects in ESCd and JAr cells and also demonstrated the highest viral replication rates in all the cell types tested, including Vero cells. The increased viral replication rates support, but certainly do not prove, the idea of mammalian host adaptation, also demonstrated by another research group where the Uganda strain produced significantly higher viral replication rates in mammalian cells (Huh7, Vero), but not in mosquito cells (C6/36, Aag2) [16]. Furthermore, the lytic effects of the Uganda strain may not reflect a real-world situation, as another study has demonstrated that high cell culture passage numbers can increase the ability of another mosquito-borne flavivirus (e.g., Japanese encephalitis virus) to lyse its host cells [62]. To this end, the addition of AF strains with a low passage history (Nigeria and Senegal strains) has strengthened our study, which indicates that trophoblast lysis is severe following infection with the highly passaged Uganda strain as well as by the low passage Nigeria and Senegal strains.

A limitation to our study was the relatively short lifespan of the ESCd culture. While it became obvious that the AF strains more rapidly lysed the ESCd, there was still significant induction of cell lysis after 60 h PI with the AS strains (Fig 2A and 2B). It is challenging to conclude if this cell lysis was exclusively induced by the virus or if there was a decline in overall culture integrity/stability that in turn made the cells more susceptible to the virus. In fact, at 72 h PI (equivalent to 7 d BAP) the mock infected controls were susceptible to mechanical disruption (cell pealing) during medium changes, fixing and staining. We suspect that this natural decline in the ESCd cultures was to some extent influencing the rate of CPE seen any time after 48 h PI. Furthermore, there was no evidence of cell lysis in JAr cells, a cell line that can be propagated indefinitely, after infection with the AS strains of ZIKV even at 72 h PI.

While both placental models, ESCd and JAr cells, clearly demonstrate the different lytic effects that AF and AS ZIKV strains have on trophoblast cells, these monoculture models do not exactly mimic the ZIKV infection pattern a multicellular in vivo placenta might encounter. Therefore, there may be confounding factors, including the maternal immune system and viral trafficking mechanisms, influencing how ZIKV targets trophoblast cells in vivo. The use of non-human primates might help to elucidate if the strain specific differences demonstrated in this study are evident in an in vivo system.

Our experiments have also provided a possible insight as to why JAr cells have been shown by other research groups to be highly susceptible to ZIKV [32, 44, 45], while in our previous study they were not [38]. It appears that JAr cells that had been dispersed by non-enzymatic means were less susceptible to ZIKV than those that had been dissociated by trypsin treatment. Presumably trypsin is able to remove cell surface components protective against ZIKV infection, whereas such components are retained when non-enzymatic dispersion is employed. For example, overexpression of IFITM3, an interferon-inducible transmembrane protein, has been reported to prevent ZIKV infection and ZIKV induced cell lysis in HeLa cells [63]. Therefore, if trypsin were to remove or alter the function of the IFITM3 transmembrane protein, it is likely that this could contribute to the increased susceptibility of JAr cells to ZIKV infections.

In conclusion, our study shows that both ESCd and the choriocarcinoma cell line JAr were vulnerable to lysis by AF strains of ZIKV, whereas contemporary AS strains, though equally infectious and able to replicate similarly, were significantly less lytic. On this basis, we present a non-intuitive but plausible explanation for the apparent lack of association between infection with AF lineage ZIKV strains and adverse pregnancy outcomes: in short, infection with the AF lineage strains may be so rapidly destructive to the developing placenta that preclinical, early pregnancy losses obscure our ability to make such associations.

## Supporting information

S1 Table Description of the ZIKV strains used in this study. (DOCX)

S1 Fig Immunofluorescence assay-based detection of ZIKV in ESCd at 24 h PI. (A) ESCd were infected with each strain of ZIKV at 1 MOI. The cells were fixed at 24 h PI and the abundance of ZIKV antigen was detected by using human anti-ZIKV polyclonal antibodies (red). Nuclei were counterstained with DAPI (blue). Representative images are shown at high and low magnifications. Scale bars are 200 μm. (B) ZIKV infected cells were counted from six representative fields. Numbers of cells per field infected with AF strains are shown in red and those infected with AS strains are shown in blue. The number of ZIKV positive cells was significantly higher in the AF Uganda infected cultures when compared to all other strains. Significance was determined by a one-way ANOVA (***p < 0.001).
(DOCX)

S2 Fig Relative susceptibility of JAr and Vero cells to AF and AS ZIKV strains. JAr and Vero cells were infected with each ZIKV strain at 1 MOI and fixed 72 h PI. Respective mock infected controls are shown below. Induction of cell death was severe in JAr cells after infection with all three AF strains, while no evidence of cell death was present after infection with the AS strains (top two rows). Similar CPE, demonstrated by the amount of cell death, became evident when Vero cells were infected with AF and AS strains (bottom two rows). A solid line separates the Nigeria strain from the other five strains because this virus was analyzed separately with a slightly higher seeding density. Scale bars are 1 mm.
(DOCX)

S3 Fig Growth curve analyses of three AF and three AS ZIKV strains in ESCd, JAr, and Vero cells. Cells were infected with the ZIKV strains at a 0.1 MOI. Cell supernatants were harvested at the indicated time points for titration by plaque assay in Vero cells. Growth curve analyses were performed in triplicate in at least two independent experiments. Data are representative of one independent experiment, plotted as SEM. Data obtained from Vero cells, ESCd, and JAr cells are shown by green, red, and blue curves, respectively. (A) The AF Nigeria strain produced similar viral titers in all three cell lines, whereas the AF Senegal and AF Uganda strains produced significantly higher titers in the Vero cells by 48 h PI (*p* < 0.001). Results from JAr and ESCd cells were not significantly different from each other. (B) All three AS strains produced significantly higher titers in Vero cells by 48 h PI than in ESCd and JAr cells (*p* < 0.001). Results from JAr and ESCd cells were not significantly different from each other.
(DOCX)

S4 Fig Representative plaque sizes caused by the different ZIKV strains in Vero cells. Cells were fixed at 5 days PI and agarose layers removed. To visualize the plaques, cells were stained with crystal violet. Highlighted by white rectangles are typical plaque types generated by each ZIKV strain.
(DOCX)
