# Supplementary material for: African and Asian strains of Zika virus differ in their ability to infect and lyse primitive human placental trophoblast
Source: PLoS One. 2018 Jul 9;13(7):e0200086. doi: 10.1371/journal.pone.0200086 (PMC6037361; doi:10.1371/journal.pone.0200086)
Supplement: S1 Table — (DOCX) [file pone.0200086.s001.docx]

**S1 Table Description of the ZIKV strains used in this study.**

| Strain | Year of Isolation (Location) | GenBank # | Passage History |
| --- | --- | --- | --- |
| FSS 13025 | 2010 (Cambodia) | JN860885 | 3x Vero cells |
| H_PANcdc259249 | 2015 (Panama) | KX156775 | 1x Vero cells |
| MEX I-44 | 2016 (Mexico) | KX856011 | 4x Vero cells |
| MR766 | 1948 (Uganda) | HQ234498.1 | 146x Suckling mice, 1x C6/36 |
| IB H30656 | 1968 (Nigeria) | HQ234500 | 1x SM21, 2x Vero cells |
| SEN/1984/41525-DAK | 1984 (Senegal) | KU955591 | 1x AP61, 1x C6/36, 1x Vero cells |

Passage histories were provided by BEI Resources and the WRCEVA. All six ZIKV strains were

passaged one additional time in Vero cells to produce the viral stocks used in all experiments.
