# Supplementary material for: African and Asian strains of Zika virus differ in their ability to infect and lyse primitive human placental trophoblast
Source: PLoS One. 2018 Jul 9;13(7):e0200086. doi: 10.1371/journal.pone.0200086 (PMC6037361; doi:10.1371/journal.pone.0200086)
Supplement: S1 Fig — (A) ESCd were infected with each strain of ZIKV at 1 MOI. The cells were fixed at 24 h PI and the abundance of ZIKV antigen was detected by using human anti-ZIKV polyclonal antibodies (red). Nuclei were counterstained with DAPI (blue). Representative images are shown at high and low magnifications. Scale bars are 200 μm. (B) ZIKV infected cells were counted from six representative fields. Numbers of cells per field infected with AF strains are shown in red and those infected with AS strains are shown in blue. The number of ZIKV positive cells was significantly higher in the AF Uganda infected cultures when compared to all other strains. Significance was determined by a one-way ANOVA (***p < 0.001). (DOCX) [file pone.0200086.s002.docx]

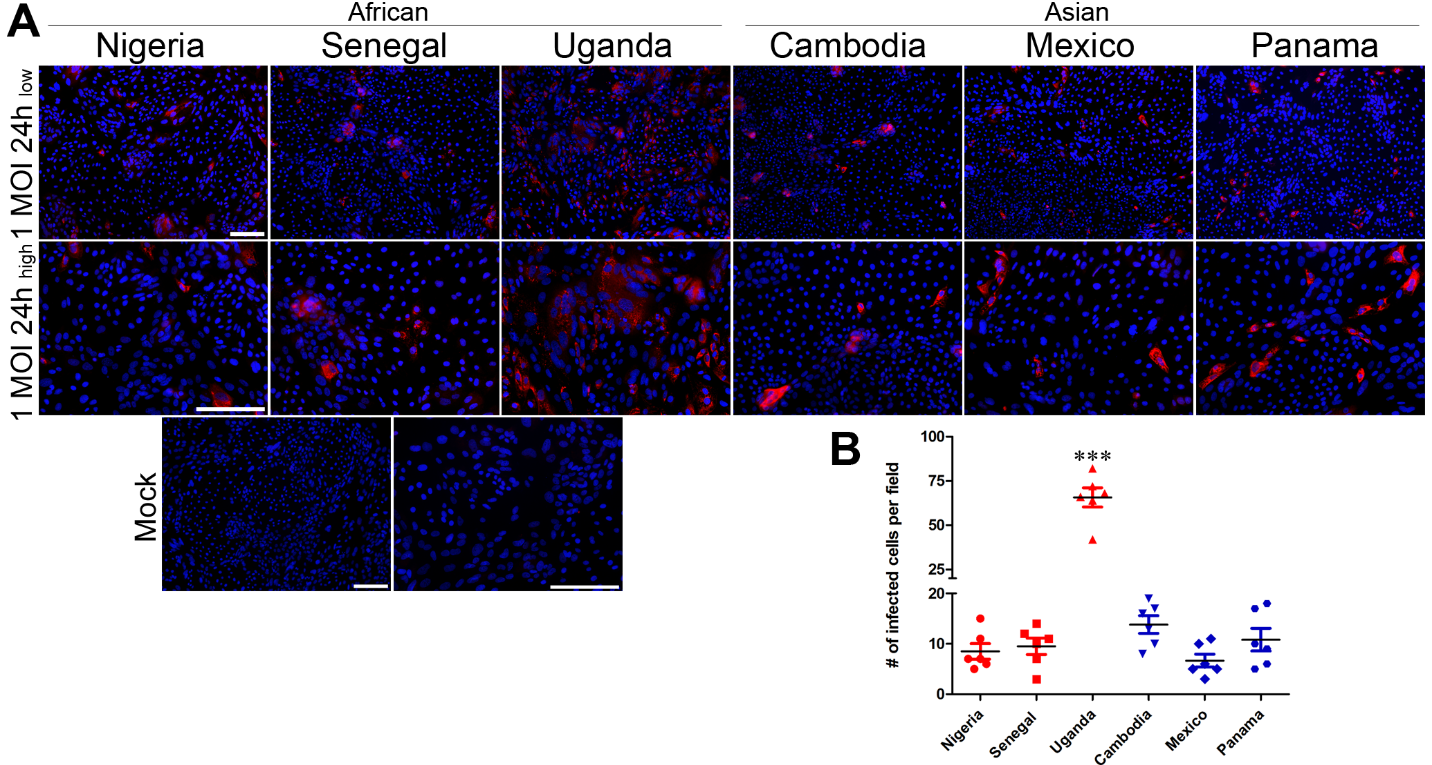


**S1 Fig Immunofluorescent detection of ZIKV in ESCd at 24 h PI.**

(A) ESCd were infected with each strain of ZIKV at 1 MOI. The cells were fixed at 24 h PI and the abundance of ZIKV antigen was detected by using human anti-ZIKV polyclonal antibodies (red). Nuclei were counterstained with DAPI (blue). Representative images are shown at high and low magnifications. Scale bars are 200 μm. (B) ZIKV infected cells were counted from six representative fields. Numbers of cells per field infected with AF strains are shown in red and those infected with AS strains are shown in blue. The number of ZIKV positive cells was significantly higher in the AF Uganda infected cultures when compared to all other strains. Significance was determined by a one-way ANOVA (*** *p* < 0.001).
