# Supplementary material for: African and Asian strains of Zika virus differ in their ability to infect and lyse primitive human placental trophoblast
Source: PLoS One. 2018 Jul 9;13(7):e0200086. doi: 10.1371/journal.pone.0200086 (PMC6037361; doi:10.1371/journal.pone.0200086)
Supplement: S2 Fig — JAr and Vero cells were infected with each ZIKV strain at 1 MOI and fixed 72 h PI. Respective mock infected controls are shown below. Induction of cell death was severe in JAr cells after infection with all three AF strains, while no evidence of cell death was present after infection with the AS strains (top two rows). Similar CPE, demonstrated by the amount of cell death, became evident when Vero cells were infected with AF and AS strains (bottom two rows). A solid line separates the Nigeria strain from the other five strains because this virus was analyzed separately with a slightly higher seeding density. Scale bars are 1 mm. (DOCX) [file pone.0200086.s003.docx]

**
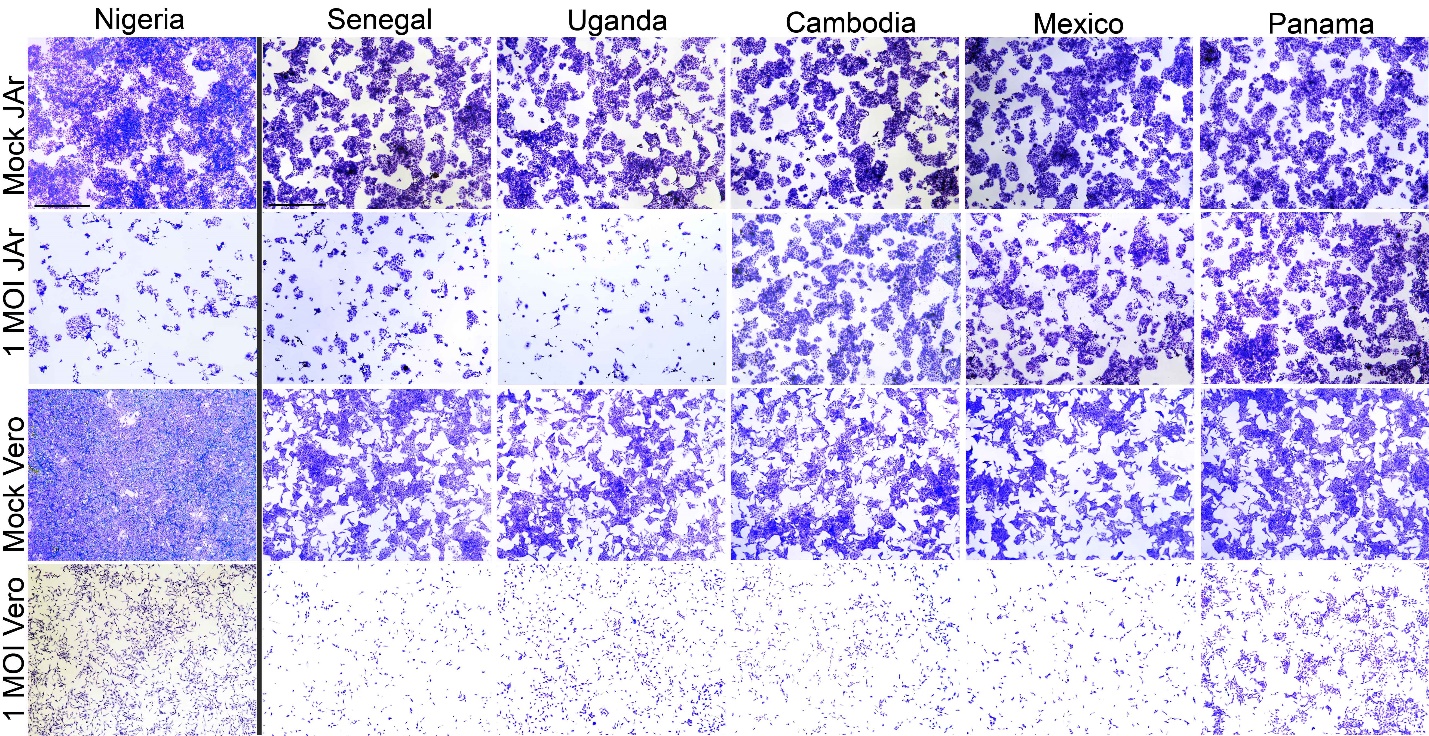
**

**S2 Fig Relative susceptibility of JAr and Vero cells to AF and AS ZIKV strains.**

JAr and Vero cells were infected with each ZIKV strain at 1 MOI and fixed 72 h PI. Respective mock infected controls are shown below. Induction of cell death was severe in JAr cells after infection with all three AF strains, while no evidence of cell death was present after infection with the AS strains (top two rows). Similar CPE, demonstrated by the amount of cell death, became evident when Vero cells were infected with AF and AS (bottom two rows). A solid line separates the Nigeria strain from the other five strains because this virus was analyzed separately with a slightly higher seeding density. Scale bars are 1 mm.
