# Supplementary material for: African and Asian strains of Zika virus differ in their ability to infect and lyse primitive human placental trophoblast
Source: PLoS One. 2018 Jul 9;13(7):e0200086. doi: 10.1371/journal.pone.0200086 (PMC6037361; doi:10.1371/journal.pone.0200086)
Supplement: S3 Fig — Cells were infected with the ZIKV strains at a 0.1 MOI. Cell supernatants were harvested at the indicated time points for titration by plaque assay in Vero cells. Growth curve analyses were performed in triplicate in at least two independent experiments. Data are representative of one independent experiment, plotted as SEM. Data obtained from Vero cells, ESCd, and JAr cells are shown by green, red, and blue curves, respectively. (A) The AF Nigeria strain produced similar viral titers in all three cell lines, whereas the AF Senegal and AF Uganda strains produced significantly higher titers in the Vero cells by 48 h PI (p < 0.001). Results from JAr and ESCd cells were not significantly different from each other. (B) All three AS strains produced significantly higher titers in Vero cells by 48 h PI than in ESCd and JAr cells (p < 0.001). Results from JAr and ESCd cells were not significantly different from each other. (DOCX) [file pone.0200086.s004.docx]

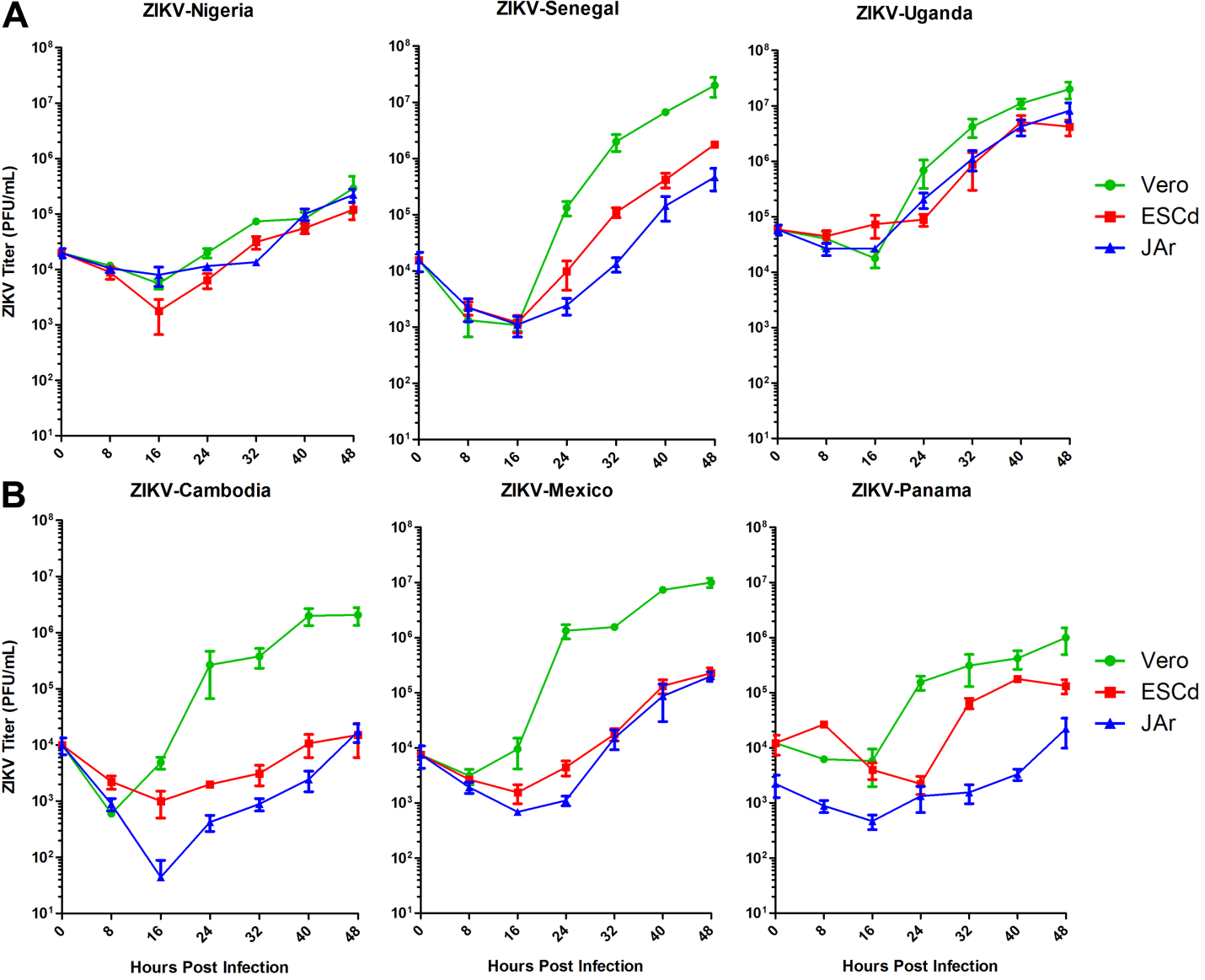


**S3 Fig**  **Growth curve analyses of three AF and three AS ZIKV strains in ESCd, JAr, and Vero cells.** Cells were infected with the ZIKV strains at a 0.1 MOI. Cell supernatants were harvested at the indicated time points for titration by plaque assay in Vero cells. Growth curve analyses were performed in triplicate in at least two independent experiments. Data are representative of one independent experiment, plotted as SEM. Data obtained from Vero cells, ESCd, and JAr cells are shown by green, red, and blue curves, respectively. (A) The AF Nigeria strain produced similar viral titers in all three cell lines, whereas the AF Senegal and AF Uganda strains produced significantly higher titers in the Vero cells by 48 h PI (*p* < 0.001). Results from JAr and ESCd cells were not significantly different from each other. (B) All three AS strains produced significantly higher titers in Vero cells by 48 h PI than in ESCd and JAr cells (*p* < 0.001). Results from JAr and ESCd cells were not significantly different from each other.
