# Supplementary material for: African and Asian strains of Zika virus differ in their ability to infect and lyse primitive human placental trophoblast
Source: PLoS One. 2018 Jul 9;13(7):e0200086. doi: 10.1371/journal.pone.0200086 (PMC6037361; doi:10.1371/journal.pone.0200086)
Supplement: S4 Fig — Cells were fixed at 5 days PI and agarose layers removed. To visualize the plaques, cells were stained with crystal violet. Highlighted by white rectangles are typical plaque types generated by each ZIKV strain. (DOCX) [file pone.0200086.s005.docx]

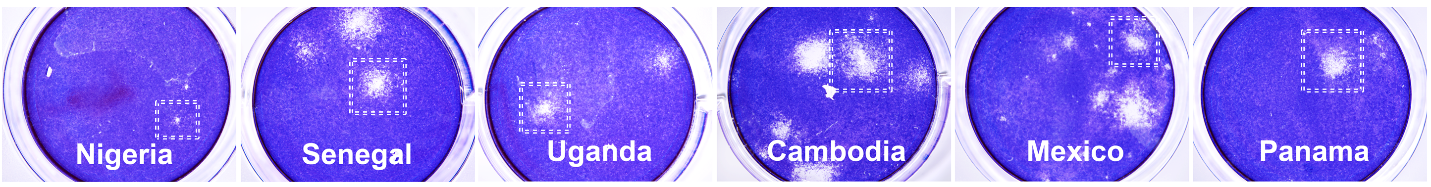


**S4 Fig**  **Representative plaque sizes caused by the different ZIKV strains in Vero cells.** Cells were fixed after 5 days PI and agarose layers removed. To visualize the plaques, cells were stained with crystal violet. Highlighted by white rectangles are typical plaque types generated by each ZIKV strain.
